# The role of personal self-regulation and regulatory teaching to predict motivational-affective variables, achievement, and satisfaction: a structural model

**DOI:** 10.3389/fpsyg.2015.00399

**Published:** 2014-04-21

**Authors:** Jesus De la Fuente, Lucía Zapata, Jose M. Martínez-Vicente, Paul Sander, María Cardelle-Elawar

**Affiliations:** ^1^Department of Psychology, School of Psychology, University of AlmeríaAlmería, Spain; ^2^Education & Psychology I+D+i, University of AlmeríaAlmería, Spain; ^3^Department of Psychology, Cardiff Metropolitan UniversityCardiff, UK; ^4^Education, Arizona State UniversityPhoenix, AZ, USA

**Keywords:** personal self-regulation, regulatory teaching, teaching–learning process, empirical model, higher education

## Abstract

The present investigation examines how personal self-regulation (*presage variable*) and regulatory teaching (*process variable of teaching*) relate to learning approaches, strategies for coping with stress, and self-regulated learning (*process variables of learning*) and, finally, how they relate to performance and satisfaction with the learning process (*product variables*). The objective was to clarify the associative and predictive relations between these variables, as contextualized in two different models that use the presage-process-product paradigm (the Biggs and DEDEPRO models). A total of 1101 university students participated in the study. The design was cross-sectional and retrospective with attributional (or selection) variables, using correlations and structural analysis. The results provide consistent and significant empirical evidence for the relationships hypothesized, incorporating variables that are part of and influence the teaching–learning process in Higher Education. Findings confirm the importance of interactive relationships within the teaching–learning process, where personal self-regulation is assumed to take place in connection with regulatory teaching. Variables that are involved in the relationships validated here reinforce the idea that both personal factors and teaching and learning factors should be taken into consideration when dealing with a formal teaching–learning context at university.

## Introduction

In Higher Education, *teaching and learning processes* make up a single binomial for the purpose of preparing students and ensuring their success. Currently, higher education is undergoing changes due to the need for quality education. This new system is based on teaching for competencies, meaning new demands for both students and teachers, and restructuring the teaching–learning process itself (Entwistle, 1988; Entwistle and Peterson, 2004a; Elliot and Dweck, 2007). It becomes essential for students to have an active role in constructing their own learning, while the teacher becomes responsible for advising and assisting students throughout the process. Thus, students have a bigger workload, they must be more responsible and they must be consistently more independent in their learning process. These changes affect how they ought to approach the educational situation, taking into account affective-motivational variables, cognitive variables and strategic variables alike (Paoloni, 2014). This new scenario can become a stressful context for students, due to its novelty and to the demands of competency-based learning (Law, 2007; Hamaideh, 2011).

### Models of Teaching–Learning Processes as Research Heuristics in Higher Education

#### The 3P Model

The study of university teaching–learning processes has been approached using different heuristics. The *3P model* (Biggs, 2005) is an *important heuristic* of study processes, suitable for contextualizing the study of relationships between different variables within university learning. It is structured along three moments of time, corresponding to the three components for which the model is named (Biggs, 1988, 1993):

(1) *Presage or prognostic*: Presage variables are variables associated with a time prior to beginning the teaching–learning process. These variables can be grouped into characteristics that depend on the students, and characteristics that depend on the teaching context. For example, student personality variables, cognitive styles, and self-regulation would be variables related to the learning process. The level of stress in the environment would be related to the teaching process.
(2) *Process*: Refers to how learning tasks are undertaken, that is, the way that the student processes and carries out the task in a specific context. The learning activities that the student undertakes are the main factor in this phase. This moment is very important in the Biggs (2005) model, constantly pointing to what students do in order to learn. These student activities will depend on their reflection, including how they perceive themselves, and how they perceive the task and the context in which it takes place. Biggs (1987) calls this reflection “meta-learning”; it requires a certain amount of metacognition and constitutes the more or less conscious awareness of and control over one’s own learning. As a function of this, students use different learning approaches in going about their activities (Elias, 2005). Learning approach, coping strategies, and self-regulated learning would be examples of variables belonging to the student’s learning process.
(3) *Product*: This includes learning outcomes. When we speak of quality learning, we must keep in mind the nature of all kinds of outcomes. Three types stand out: (a) Quantitative: Quantity of information, data and concrete skills acquired; (b) Qualitative: Structure/Complexity of thought and transfer of the knowledge that has been developed; (c) Affective: Student satisfaction and engagement in the process. Therefore, performance and satisfaction with learning would be examples of variables from this phase.

#### The DEDEPRO Model

De la Fuente et al. (2006, 2014) make contributions to Biggs’ 3P model from an interactive perspective of the teaching–learning process, framed within the new context of the European Higher Education Area (EHEA). This has resulted in creation of the DEDEPRO model (De la Fuente and Justicia, 2007) as a *complementary heuristic* along with the former model, able to guide research on variables that interact within the university teaching–learning process, especially while the teaching process is under way (in “development”). DEDEPRO is an acronym for the phases of Design-Development-Product. Regulation of teaching and learning are assumed, and are expressed in terms of macro-regulation and micro-regulation (De la Fuente et al., 2005). This model seeks to integrate conceptual contributions from regulation, keeping in mind both the learning process and the teaching process. In essence, the model assumes that self-regulated learning should be connected to regulatory teaching, as a type of effective teaching (De la Fuente and Martínez-Vicente, 2004; De la Fuente et al., 2005, 2006; De la Fuente and Justicia, 2007; De la Fuente, 2011).

The DEDEPRO model adopts characteristics from Biggs’s (2001) 3P model and from the Zimmerman and Kitsantas (1997) model. The DEDEPRO phases correspond to those established by Zimmerman (2002, p. 67), applied to the teaching–learning process: “preparatory or design phase, execution or development phase and reflection or product phase.” The conceptual model arises from different theoretical assumptions that are based on the empirical evidence of studies carried out in university and non-university stages of education (De la Fuente and Martínez-Vicente, 2004). In a recent report, De la Fuente et al. (2014) offer empirical evidence regarding the four possible types of interactive relations between the student’s level of personal self-regulation and the level of regulatory teaching, as well as their effects on self-regulated learning, performance, and academic behavioral confidence in university students. These combinations are shown in **Table 1**. The main contribution of this model is the concept of *regulatory teaching.*

**Table 1:** Types of relations between levels of variables in the DEDEPRO model, in the context of the 3P model (reproduced with permission).

Type	Presage	Process (design and regulated development)		Product	
Level	Personal self-regulation	Regulatory teaching	Self-regulated learning	Performance	Academic behavioral confidence
4°	High	High	High	High	High
3°	High	Low	Moderate/high	Moderate/high	Moderate/high
2°	Low	High	Moderate/low	Moderate/low	Moderate/low
1°	Low	Low	Low	Low	Low

### Personal Self-Regulation as a Presage Variable of Learning

*Personal self-regulation* refers to the capacity or ability to control our own thoughts, emotions, and actions. Within this theoretical framework, Brown (1998, p. 62) defines self-regulation as a person’s ability to “plan, monitor and direct his or her behavior in changing situations.” In essence, this model adopts the self-regulation postulates of Zimmerman (2002), by defining moments of planning, control and thoughtful evaluation of one’s action. It is therefore a *meta-skill* or behavior management skill, used in any context, and includes, for example, setting goals (before taking action), self-monitoring and persisting in one’s effort (during the action) and final reflection and learning from mistakes (after taking action).

We can therefore affirm that personal self-regulation is a vital process that allows people to behave adequately, carry out tasks properly, and abstain from activities that may be harmful to them (Baumeister et al., 1994; Baumeister and Heatherton, 1996). Self-regulation is used in a number of processes including the regulation of emotions, thoughts, and actions for physical or behavioral control or restraint (Baumeister et al., 1998; Vohs et al., 2008).

Personal self-regulation, as a psychological variable that is closely tied to subjects’ personal development competencies, has attracted interest in the sphere of educational psychology. Prior studies have shown that self-regulation has a significant role in health as well as in success, whether academic or work-related (Karoly et al., 2005; Vancouver and Scherbaum, 2008). We can think of the process of self-regulation as having a personal, behavioral, and contextual nature (Bandura, 1986; Torrano-Montalvo and González-Torres, 2004), adding goals as a key factor (Latham and Locke, 1991, 2007; Winne, 2004). Taking personal regulation as a *presage* variable in the sphere of educational psychology, De la Fuente and Cardelle-Elawar (2011, p. 3) define it as a *presage* student variable “that determines the level of effort that students will sustain in the process of active learning for the completion of a given task.”

### Learning Approaches, Coping Strategies, and Self-Regulated Learning as Learning Process Variables

#### Approaches to Learning

The origin and development of *learning approaches* stem from a series of research studies that, although carried out in different socio-cultural and educational contexts and with different methodologies, have concurred in identifying (initially) three approaches to learning: deep approach, surface approach and achievement approach (Marton and Säljö, 1976; Biggs, 1994; Entwistle, 2000; Entwistle and Peterson, 2004b). Marton and Säljö (1976) took a qualitative perspective for their research, while Biggs (1994) and Entwistle (2000) performed studies from a more quantitative perspective. But both research traditions fall under what we call student-perspective research, that is, the focus is on learning from the student’s perspective, in order to understand the intentions, interests, strategies, and motives that lead students to take on academic tasks and act in a certain way in a specific situation.

Within the 3P model, Biggs (1989, 1990, 2001, 2006) indicates that the three prototypical approaches to learning that are most important in students’ learning processes are the Surface, Deep, and Achievement approaches. But in Biggs’ questionnaire revalidation, he maintains a dual structure of surface vs. deep (Biggs, 2001), and these two learning approaches are what we measure in this investigation. Students who adopt a *surface approach* are motivated instrumentally, pragmatically or extrinsically, and their main purpose is to meet the course requirements with the least effort. Thus, learning becomes a balancing act between avoiding failure and not working too hard. The most appropriate *strategies* for this purpose are those that are limited to the essential (mechanical), focusing on literal, specific aspects of the tasks. This surface strategy, or reproductive strategy, is indifferent to any interrelationships that may exist between task components, such that the task is not perceived as a unified whole. Conversely, the affective orientation of students that adopt a *deep approach* is intrinsic motivation to understand and to enjoy learning. Thus, they adopt strategies that are most likely to help them satisfy their curiosity and their search for inherent meaning in the task.

In recent decades there has been a large volume of research on approaches to learning (Sander et al., 2012). In general, the deep approach is a good predictor of academic success and the surface approach is associated with poorer results (Bernardo, 2003; English et al., 2004; Snelgrove, 2004).

#### Coping Strategies

The concept of stress has been studied at length, and there are many authors who examine it and seek to define it. Holroyd and Lazarus (1982, p. 843) define *coping* as “cognitive and behavioral efforts to master, reduce, or tolerate the internal and/or external demands that are created by the stressful transaction.” Lazarus (1991, p. 112) defines coping as “cognitive and behavioral efforts to manage specific external or internal demands (and conflict between them) that are appraised as taxing or exceeding the resources of a person.”

There are a variety of *coping strategies* that have been proposed by researchers in order to understand the discrepancies in how individuals act when dealing with stressful situations (Lazarus and Folkman, 1986; Hobfoll and Schröeder, 2001; DeLongis et al., 2010). There are diverse definitions of *strategies for coping with stress,* but in general terms, we can say that the concept refers to behavioral and cognitive efforts that a person makes in order to deal with stress. Coping strategies in the context of Educational Psychology are more related to academic stress and specifically to one of its main stressors, tests (Piemontesi and Heredia, 2009; Day et al., 2013). Fewer studies have been carried out in this field, but relationships have been found between coping strategies and academic performance (Cohen et al., 2008) and student gender (De la Fuente et al., 2013a). In addition, students’ levels of stress have been studied in conjunction with the coping strategies they use (Ticona et al., 2010).

Lazarus and Folkman (1986) consider one distinction to be extremely important: the difference between coping that is directed toward handling or modifying the problem, and coping that aims to regulate the emotional response that the problem brings about. The first is referred to as *problem-focused coping* and the second as *emotion-focused coping* (Lazarus and Folkman, 1984). In general, the former are more likely to appear when the harmful or stressful conditions are appraised as subject to change. Emotion-focused strategies are more likely to appear when the appraisal indicates that nothing can be done to modify the threatening conditions of the environment (Lazarus and Folkman, 1986):

(1) *Emotion-focused ways of coping*: The literature mentions a large number of such ways of coping, but we can divide them into two large groups: (a) Cognitive processes dedicated to decreasing the degree of emotional discomfort, including strategies such as avoidance, minimization, distancing oneself, selective attention, positive comparisons, and finding positive value in negative events; (b) Cognitive strategies that seek to increase the degree of emotional discomfort; some persons need to feel really bad before they can come to feel better; in order to find comfort they need to first experience intense discomfort, from which they can then move on to some kind of self-punishment. In other cases, they deliberately increase their degree of emotional discomfort in order to push themselves to action, such as when athletes challenge themselves in order to compete. Examples of emotion-focused behaviors are: getting exercise, having some fun, relaxation, praying, drinking, or partying.
(2) *Problem-focused ways of coping*: These strategies are similar to those used for solving the problem; they are directed at the definition of the problem, the search for alternative solutions, consideration of these alternatives based on cost and benefit, and the selection and application of alternative(s). An objective is also involved, an analytical process directed mainly at the environment. However, these ways of coping also include strategies internal to the person. We can therefore speak of two main groups of problem-focused strategies: those that refer to the environment and seek to modify environmental pressures, obstacles, resources, procedures, etc.; and those that refer to the subject, including strategies dedicated to motivational or cognitive changes, changing one’s level of aspirations, reducing involvement of the ego, seeking different channels for gratification, developing new behavior patterns, or learning new resources and procedures. Examples of problem-focused behaviors are: making decisions, seeking help, designing a plan, reassessing the problem, and taking action toward a solution.

#### Self-Regulated Learning

The concept of self-regulated learning is receiving more and more attention, due to its fundamental importance in the teaching–learning process. Specifically, this construct refers to a self-directing process in students, transforming their mental ability into academic skills. Self-regulated learning is thus considered a proactive activity where the student takes the lead in helping himself, as well as in developing learning strategies. When defining this variable, we must bear in mind the active role of students in the learning process, the feedback given to them during this process, and the role of motivation (Zimmerman and Labuhn, 2012; Benbenutty et al., 2014). We can consider *self-regulated learning* as a learning meta-skill or a specific case of *personal self-regulation* within a given learning situation or task.

Researchers who study this variable suggest that students self-regulate, at the metacognitive, motivational, and behavioral levels, when they take an active role in their teaching–learning process (Zimmerman, 1986). All the definitions given to self-regulated learning include these three properties, allowing students to be aware of their own learning process and of the importance of improving their academic performance. But these are not the only components in the definition of this construct, we also find what are known as feedback loops during learning (Zimmerman, 1989, 2000; Winne and Hadwin, 1998; Carver and Scheier, 2000). Socio-cognitive theory emphasizes the interaction of personal, behavioral, and environmental factors (Bandura, 1997; Zimmerman, 2002). These factors normally change during learning and must be monitored, hence, self-regulation is considered to be a cyclical process. Such monitoring leads to changes in the student’s strategies, cognition, affect, and behavior. This cyclical nature is represented in Zimmerman’s three-phase self-regulation model (Zimmerman, 1998):

(1) *Forethought phase*: A phase that precedes execution and refers to processes that prepare the scenario for action, giving thought to processes that occur during learning and that affect attention and action. During this initial phase, there are two different areas: task analysis processes and self-motivation beliefs. Task analysis involves a learner’s efforts to break down a learning task into its key components. Students’ task analyses influence their goal setting and planning.
(2) *Performance control phase*: Two major classes of self-regulation processes are postulated during this phase: self-control and self-observation. The first of these processes refers to the actual use of different strategies to guide learning, such as task, cognitive, and behavioral strategies. The second process refers to specific methods to track one’s performance; metacognitive monitoring deals with informal mental tracking of one’s processes and outcomes in the performance phase, whereas self-recording indicates creating formal records of the learning process and/or outcomes.
(3) *Self-reflection phase*: This phase takes place after execution; students respond to the efforts they have made, with greater effort compensating for fewer self-regulation processes throughout the different phases. Students come to learning situations with different goals and different levels of self-efficacy for attaining them. While monitoring execution, they implement learning strategies, which then affect motivation and learning. Two types of processes occur during the self-reflection phase: self-judgments and self-reaction. Self-judgments refer to self-evaluations of the effectiveness of one’s learning performance and causal attributions regarding one’s outcomes. Learners’ self-judgments are linked to two key forms of self-reactions: self-satisfaction and adaptive inferences. Self-satisfaction reactions refer to perceptions of satisfaction or dissatisfaction, and their associated affect, in regard to one’s performance. These emotions can range from elation to depression. A closely associated type of self-reaction involves adaptive or defensive inferences, which refer to conclusions about whether and how a learner needs to alter his or her approach during subsequent efforts to learn. These self-reactions influence forethought processes in further problem-solving efforts, thus completing the self-regulatory cycle (Zimmerman and Labuhn, 2012).

### Academic Performance and Satisfaction as Product Variables of the Learning Process

#### Academic Performance

Every teaching–learning process aims toward a certain product, with certain objectives and purposes that are to result in the student learning a specific subject matter. This product is called *academic performance*. Performance has been defined and categorized by different authors. Most research has analyzed performance based on a single overall qualification. This tendency to reduce the outcome of learning to a single grade has become one of the main criticisms of research on academic performance. Biggs (1999, 2001) proposes an alternative to address the problem of reducing academic performance, describing the product of teaching–learning through different outcomes classified as quantitative, qualitative, and affective (satisfaction).

We have seen that Biggs’ proposal is not the only way to rectify the simplistic view of academic performance. De la Fuente et al. (2004) base academic performance on a compendium of competencies: *conceptua*l (grades achieved on exams), *procedural* (class attendance and lab work), and *attitudinal* (class participation and voluntary efforts). Academic performance has taken on greater importance in educational research in recent decades, with many variables being studied for their influence on the academic performance of university students.

#### Satisfaction with the Learning Process

The third dimension of learning outcomes is affective performance (Biggs, 2001), referring in this case to *satisfaction* with the learning process and with the result obtained. Academic satisfaction is concerned both with one’s performance (in a subject, course, or degree program), and with the characteristics of the teaching process. In both cases, a simplistic definition of satisfaction refers to the degree that the student’s expectations were met, with regard to the teaching process or performance. Affective performance has been studied the least, but Locke (1976) proposed a rather widely accepted definition. According to this author, satisfaction is a pleasurable emotional state that results from students’ perception that certain activities are making it possible to reach values that are important to them, being consistent with their needs.

### Regulatory Teaching as a Process Variable of Effective Teaching

*Regulatory teaching* is a process variable in the DEDEPRO model (De la Fuente and Justicia, 2007; De la Fuente, 2011). It is characteristic of *effective teaching* (Roehrig and Christesen, 2010; Roehrig et al., 2012), incorporating the following aspects:

(1) *Good organization,* with flexibility, to promote student engagement. An effective teacher is a good organizer, anticipating problems, and seeking planning alternatives (Roehrig and Christesen, 2010). Similarly, this teacher plans for success, using a variety of instructional strategies in each lesson. When comparing new teachers to expert teachers, the experts differ in the complexity of knowledge elaboration, in how they respond automatically to planning-related situations, in the decision-making process: student groupings, selection of work material, and innumerable decisions involved in adaptation to the class group (Corno, 2008), and in scheduling of tasks. When teachers plan well and use good methods, the students are more involved (Roehrig and Christesen, 2010). Fundamentally, this practice refers to promoting students’ metacognition and knowledge about monitoring their own cognitive processes. *Metacognition* is the highest order level of thought, and it can be implemented in students’ learning through teaching. The highest level of *cognitive engagement* (the teaching of thought) *is a good level of emotional engagement* (positive atmosphere), in interaction with other actions that show *behavioral engagement* (class management), how one educates to involve others (Fredricks et al., 2004). Teacher behaviors that predict self-regulated learning are modeling and activities in the zone of proximal development. These practices for helping students reveal the content and skills to which self-regulated thinking, behavior and affect are to be applied (Zimmerman, 1989).
(2) *Promotion of self-regulated learning.* Promoting self-regulation and motivation in students requires activities where students must make use of *planning* and *self-monitoring* (Perry et al., 2006). Self-regulation incorporates the four dimensions we have alluded to: teaching in a positive climate, motivation of students, providing instructional support and assistance and modeling one’s planning and control of the process. Self-regulation is a multidimensional aspect of effective teaching, whereby clear expectations are put forward, automated routines are established, and students are redirected and assisted as they complete their academic tasks.
(3) Also required is the establishment of *good teacher-student relations* (Buyse et al., 2008), as well as the opportunity to practice self-regulating behaviors. Teachers that help their students exercise self-regulation possess the most salient aspects of engagement with the class (Raphael et al., 2008). Some research studies have established how good teachers monitor, prevent and redirect behavior, and how they establish routines, as the most important aspects of this teaching behavior.

Effective teaching refers to teaching efficacy, involving adequately structured teaching and assistance in order to facilitate and induce self-regulated learning (Kramarski and Michalsky, 2010). By this we refer to the idea that the teacher should know how to “externally regulate” the learning process in order to contribute to students’ “self-regulation” of the learning process; thus, a strong component of self-regulation is required when teaching (Randi, 2004). De la Fuente and Justicia (2007) understand that a teaching process is regulatory when the activities of teaching, learning, and assessment are intrinsically interrelated for the achievement of autonomous, constructive, cooperative and diversified learning, creating a predictable teaching–learning scenario. Some teacher behaviors clearly exemplify this variable: presenting a plan of work for a certain period of time, explaining the goals and deadlines for each task, helping students set objectives before they begin a task, etc. This type of regulation in teaching is produced at both levels of self-regulation, that is, it is an equally valid principle for learning specific things (micro-regulation) and for learning as a whole (macro-regulation).

De la Fuente and Justicia (2007) hypothesize a lack of regulation in teaching and learning. This may be due to the teacher not explaining important informational elements at different moments of the teaching–learning process (design and development of the syllabus), such that students are unable to make decisions about how they should undertake their learning. This in turn leads to a lack of correct decisions about the design and development of the learning process, students learning in an unregulated fashion, and hence, poorer performance than what they potentially could have. For this reason, as we mentioned above, *explicit activities* must be carried out with regard to the teaching process, through different continuous regulation devices (Luo, 2000; Xin et al., 2000), in order to improve learning processes and student’s self-regulation thereof. Some of the teaching strategies that could be implemented are: (diagnostic and process) assessment, information supplied to students about the teaching process and the structuring of learning activities, and stimulation of self-regulation in students. We must not forget the *facilitating role* of regulatory teaching in self-regulation of learning. As some authors have already commented (Lindblom-Ylänne et al., 2011), research on regulatory teaching is scarce, and the present study offers one way of moving forward in the study of this variable, opening up an area that has been practically sealed off.

### Aims and Hypotheses

The current study addresses a broad range of different cognitive-motivational variables with the objective of building models to examine the joint effect of personal self-regulation and regulatory teaching, with other process variables, on undergraduate students’ performance and satisfaction with learning (De la Fuente et al., 2011). Some recent studies have reached an interactive conception of the teaching learning process and an important interaction between students’ individual characteristics and their learning outcomes (De la Fuente et al., 2014).

Based on the evidence from the above literature review, this investigation has two objectives: (1) To build a correlational and structural empirical model of consistent relationships that establish conceptual relations between the *learning process* variables: determining how student presage variables (personal self-regulation) relate to process variables (coping strategies, approach to learning, self-regulated learning strategies) and product variables (performance and satisfaction); (2) In complementary fashion, to build another empirical correlation and structural model with the *teaching process* variables: determining how process variables (regulatory teaching) are related to and interact with these student presage, process, and product variables.

**FIGURE 1:**
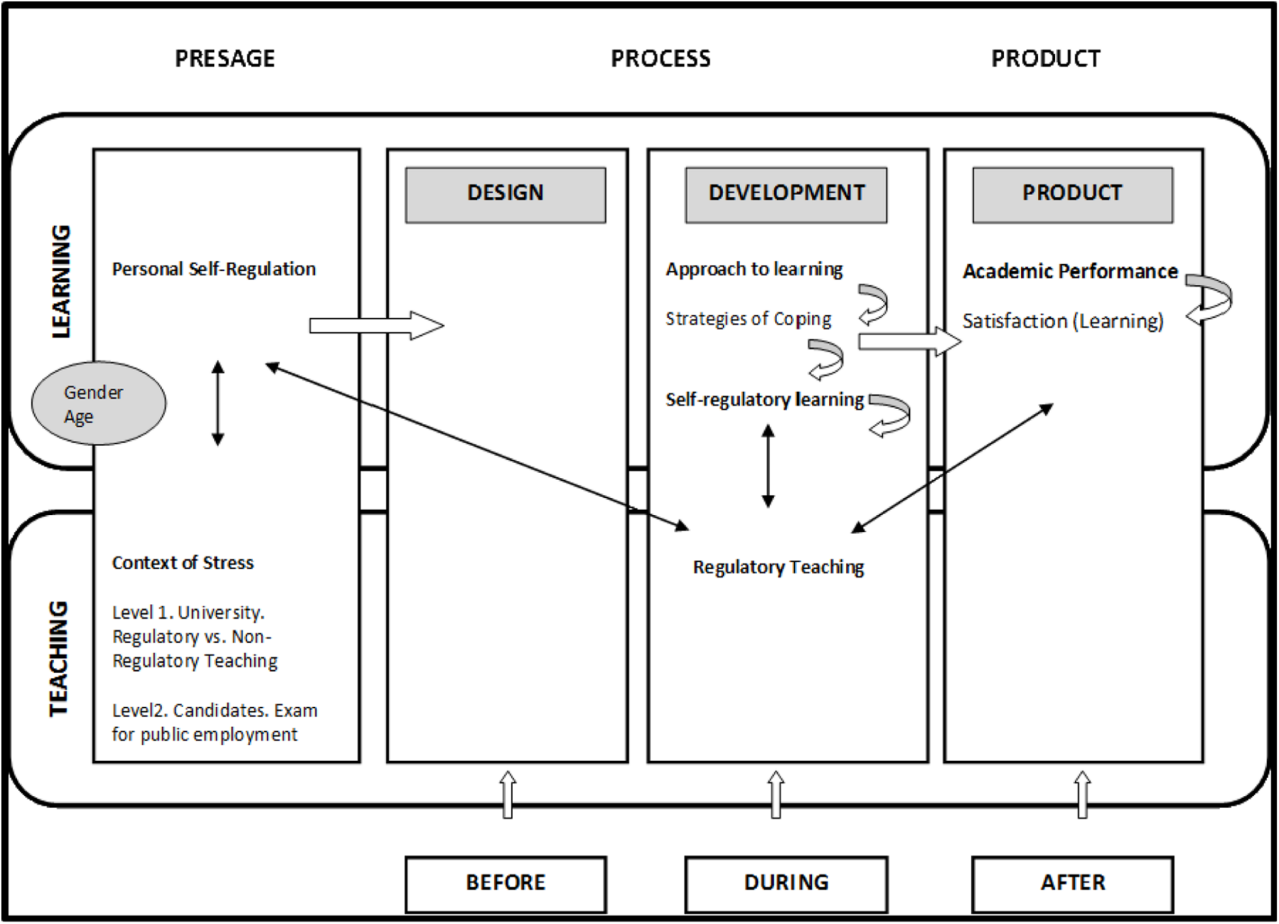
**Study variables integrated into the DEDEPRO model**.

We expect to find a structural model that validates our proposed conceptual relationships: (1) *Personal self-regulation,* especially, and goal-setting and perseverance will have a significant differential relationship with the types of *learning approaches* and *coping strategies,* and these in turn with *self-regulated learning,* which will ultimately determine *mean performance* and *satisfaction with learning.* (2) *Regulatory teaching* will also have an essential role in these relations, having a positive effect on the previous relationships mentioned. The variables that form part of this research are represented in **Figure 1**.

## Materials and Methods

### Participants

A total of 1101 students participated in the study. Of the university students, 48.3% were pursuing a degree in Psychology, and 12.1% in School Psychology (Psychopedagogy). The mean age was 23.08 years (SD = 4.4) with ages ranging from 19 to 49. Men represented 9.3% and women 59.4%, while an additional 31.3% did not provide this information when completing the questionnaires.

### Instruments

We present the measures in the same order as the variables (presage, process, and product variable) for easier understanding.

#### Learning Process

##### Presage variable

*Personal self-regulation* was measured using the *Short Self-Regulation Questionnaire SSRQ* (Miller and Brown, 1991). It has already been validated in Spanish samples (Pichardo et al., 2014), and possesses acceptable validity and reliability values, similar to the English version. The Short SRQ is composed of four factors (goal setting-planning, perseverance, decision-making, and learning from mistakes) and 17 items (all of them with saturations greater than 0.40), with a consistent confirmatory factor structure (Chi-Square = 250.83, df = 112, CFI = 0.90, GFI = 0.92, AGFI = 0.90, RMSEA = 0.05). *Internal consistency* is acceptable for the total of questionnaire items (α = 0.86) and for the factors of goal setting-planning (α = 0.79), decision-making (α = 0.72) and learning from mistakes (α = 0.72). However, the perseverance factor (α = 0.63) showed low consistency. *Correlations* have been studied between each item and its factor total, between the factors, and between each factor and the complete questionnaire, with good results for all, except for the decision-making factor, which had a lower correlation with other factors (range: 0.41–0.58). The correlations between the original version and the complete version, and between the original and the short versions with a Spanish sample (complete SRQ with 32 items and short SRQ with 17 items) are better for the short version (short-original: *r* = 0.85 and short-complete: *r* = 0.94, *p* < 0.01) than for the complete version (complete-original: *r* = 0.79, *p* <0.01).

##### Process variables

*Learning approach* was measured with the *Revised Two-Factor Study Process Questionnaire, R-SPQ-2F* (Biggs et al., 2001), in its Spanish validated version (Justicia et al., 2008). It contains 20 items on four subscales (Deep Motive, Deep Strategy, Surface Motive, and Surface Strategy), measuring two dimensions: Deep and Surface learning approaches, respectively. Students respond to these items on a 5-point Likert-type scale ranging from 1 (rarely true of me) to 5 (always true of me). The Spanish version showed a confirmatory factor structure with a second factor structure of two factors (Chi-Square = 2645.77, df = 169, CFI = 0.95, GFI = 0.91, AGFI = 0.92, RMSEA = 0.07) that also yielded acceptable reliability coefficients (Deep, α = 0.81; Surface, α = 0.77), similar to the study by the original authors.

The *coping strategies* variable was measured using the *Escala de Estrategias de Coping* (EEC) [Coping Strategies Scale], in its original version (Chorot and Sandín, 1987, 1993; Sandín and Chorot, 2003). A total of 90 items are included where students respond to items on a 4-point Likert-type scale ranging from 0 (never use the strategy) to 3 (always use the strategy). The scale was constructed according to theoretical-rational criteria, taking as its basis the questionnaire by Lazarus and Folkman (1984) and the coping assessment studies by Moos and Billings (1982). De la Fuente (2014) carried out a validation study with the original EEC (Chorot and Sandín, 1987) in a Spanish sample. EEC-R comprises two dimensions structured along 13 factors: coping focused on emotions and coping focused on the problem. The scale showed a factor structure with adequate fit indices (Chi-Square = 565.800, df = 48, *p* < 0.001, CF1 = 0.901, TLI = 0.912, NFI = 0.913, NNFI = 0.904, RMSEA = 0.056) and adequate internal consistency. The complete scale obtained a reliability of 0.93, with 0.93 for the first half and 0.90 for the second half (Cronbach alpha). The Spearman–Brown and Guttman values were 0.84 and 0.80 respectively, for each dimension. In all cases, factors from each dimension and their reliability exceed a value of 0.80.

*Self-regulated Learning* was assessed using the IATLP Scales (De la Fuente and Martínez-Vicente, 2007). IATLP Dimension 2 (De la Fuente et al., 2012), for the assessment of SRL, is composed of 16 items that are grouped into three elements that make up SRL: planning (six items, e.g., “Before beginning any learning activity or task, I organize what I have to do, telling myself: ‘first I have to do this, then I have to do that’…”), thoughtful learning (five items, e.g., “When learning, I like to relate it to my own experience and my life”) and study techniques (five items, e.g., “I usually make notations when learning new material”). Participants respond to the items on a Likert scale from 1 (strongly disagree) to 5 (strongly agree). IATLP D2 scale showed a factor structure with adequate fit indices (Chi-Square = 281.10, df = 101, *p* < 0.001, SMSR = 0.071, GFI = 0.912, AGFI = 0.881, IFI = 0.914, MFI = 0.832, CFI = 0.913, RMSEA = 0.060; 90% CI of RMSEA = 0.052–0.069) and adequate internal consistency (IATLP Dimension 2: α = 0.87; planning: α = 0.82; thoughtful learning: α = 0.82; study techniques: α = 0.79). As for the instrument’s external validity, results are again consistent, since there are different interdependent relationships between the perceptions of variables that exist in an academic environment.

*Academic performance.* We made use of the academic-professional competency assessment model (De la Fuente et al., 2004). The competencies that enable us to practice a profession are defined as the body of integrated academic-professional knowledge for optimum fulfillment of professional requirements. Following this competency model, we took the mean scores that teachers assigned to the students at the end of a full-year subject. Total performance, on a scale of 1 to 10, is the final grade given to the student for this subject. The 10 points are a compendium of results obtained on the three levels of sub-competencies, conceptual, procedural, and attitudinal: (1) *Conceptual scores*: include all scores obtained on exams covering the conceptual content of the subject (four points); (2) *Procedural scores*: assessed from the student’s practical work covering procedural content and skills (four points); (3) *Attitudinal scores*: scores given for class participation and for optional assignments undertaken for a better understanding of the material (two points). In order to carry out the different analyses and compare the results, the different sub-competency scores were converted to an equivalent scale from 1 to 10.

*Academic satisfaction.* The IATLP scales include a scale for measuring *satisfaction* with the learning process (De la Fuente and Martínez-Vicente, 2007). The scale entitled *Satisfaction with the* Learning Process is Dimension 3 of the confirmatory model. IATLP-Dimension 3 comprises 12 items structured along two factors: meaningful learning and satisfaction with learning. The scale was recently validated in university students (De la Fuente et al., 2012), showing a factor structure with adequate fit indices (Chi-Square = 590.626, df = 48, *p* < 0.001, CF1 = 0.838, TLI = 0.839, NFI = 0.850, NNFI = 0.867, RMSEA = 0.068) and adequate internal consistency (IATL D3: α = 0.80; meaningful learning: α = 0.851; satisfaction with learning: α = 0.878).

#### Teaching Process

The IATLP scales include a scale for measuring regulatory teaching (De la Fuente and Martínez-Vicente, 2007). The scale entitled *Regulatory Teaching* is Dimension 1 of the confirmatory model. IATLP-D1 comprises 29 items structured along five factors: specific regulatory teaching, regulatory assessment, preparation for learning, satisfaction with the teaching and general regulatory teaching. The scale was recently validated in university students (De la Fuente et al., 2012) and showed a factor structure with adequate fit indices (Chi-Square = 590.626, df = 48, *p* < 0.001, CF1 = 0.838, TLI = 0.839, NFI = 0.850, NNFI = 0.867, RMSEA = 0.068) and adequate internal consistency (IATLP D1: α = 0.83; Specific regulatory teaching, α = 0.897; regulatory assessment, α = 0.883; preparation for learning, α = 0.849; satisfaction with the teaching, α = 0.883 and general regulatory teaching, α = 0.883).

We assessed students’ level of perceived *satisfaction* with the instrument *Assessment of the Teaching–Learning Process* (ATLP-S). The revalidated version of this scale (De la Fuente et al., 2012) was used to assess satisfaction with the learning process. Overall reliability for this scale is α = 0.81.

### Procedure

In the second half of September 2012 and September 2013 volunteer teachers followed a training process on strategies of regulatory teaching and self-regulated learning. In essence, this process (1) explained the DEDEPRO model (De la Fuente and Justicia, 2007), as well as (2) general and specific strategies of regulatory teaching, oriented toward promoting self-regulated learning. The latter included design strategies and strategies to make the teacher’s planning explicit to students, and specific strategies for regulatory teaching. The training specifically addressed: (1) *general behaviors* of regulatory teaching: explaining the objectives at the beginning of the class, presenting a schedule of work when beginning a new topic, posing questions before giving an explanation, etc.; (2) *specific behaviors* of regulatory teaching: explaining the objectives before doing a specific activity; think-aloud modeling, before, during and after the activity; helping students self-assess, etc.; (3) *regulatory assessment* strategies: correcting answers together with the students, in class; using a continuous assessment system, etc. The training was offered using a workshop methodology. The regulatory behavior was explained, examples of its application in class were discussed, and it was modeled by the trainer. This training process lasted 20 h. Teachers in the training group were provided with a list of teaching behaviors (one of each type that had been taught) to be implemented in their classroom during the 9 months of the academic year (De la Fuente et al., 2013b, 2014).

Information from self-reports was collected from university students in the classroom, during regular class hours, over two academic years, 2012/13 and 2013/14. For the university students, data on Presage variables (personal self-regulation) was collected during the month of November. Later, in the month of February, students voluntarily completed the scales that measure Process variables (learning approaches, coping strategies, self-regulated learning, and regulatory teaching). In the month of May–June, satisfaction with learning was assessed, and teachers of the participating classes were asked for the mean total scores for each student, as measured through continuous assessment over the academic year (Product variables). Data collection was approved by the university’s Research Ethics Committee. The data were collected and saved in a registered, protected database.

### Design and Data Analysis

This investigation, in order to address its objectives and hypotheses, made use of a cross-sectional, retrospective design, with attributional (or selection) variables, for predictive purposes. Of the 1101 initial subjects, 190 were eliminated due to either incomplete personal data or not having completed all the questionnaires needed for the correlational analyses. Pearson bivariate correlations (two-tailed; SPSS program, version 22.00) and path analysis (using the AMOS program version 22.00) were carried out.

## Results

### Bivariate Correlations

#### Personal Self-Regulation and Learning Process Variables

A significant association relationship appeared between *total self-regulation* and *learning approaches* and its components (**Table 2**). Specifically, *total self-regulation* has a positive correlation with deep approach (deep motivation and deep strategy) and a negative association with surface approach (surface motivation and surface strategy). Statistically significant relationships were found for *goals* and *perseverance* in connection with *learning approaches*, namely, positive relationships with deep approach (deep motivation and deep strategy) and negative relationships with surface approach (surface motivation and surface strategy).

**Table 2:** Correlations between *personal self-regulation* (presage variable) with other variables of learning (process variables; *n* = 911).

Dimensions and factors	Total SRQ	Personal goals	Perseverance	Decision-making	Learning from mistakes
Deep learning approach	0.312^∗∗^	0.413^∗∗^	0.278^∗∗^	0.181^∗^	0.169^∗^
Surface learning approach	-0.337^∗∗^	-0.319^∗∗^	-0.276^∗∗^	-0.286^∗∗^	-0.238^∗∗^
Emotion focused	-0.181^∗∗^	-0.081	-0.142^∗^	-0.266^∗∗^	-0.099
Problem-focused	0.086	0.149^∗^	0.105	-0.110	0.123^∗^

*^∗^p < 0.05, ^∗∗^p < 0.01.*

Pearson bivariate correlation analysis showed a negative correlation between *personal self-regulation* and *emotion-focused coping strategies*, specifically in the case of *perseverance* and *decision-making*. A positive relationship was also found for *goals* and *learning from mistakes* in connection with *problem-focused strategies*.

#### Self-Regulated Learning and Other Learning Process Variables

The *deep approach* had a positive correlation with self-regulated learning and all its factors (planned learning, thoughtful learning, and study techniques) and the *surface approach* was negatively associated in the same fashion. *Problem-focused* coping strategies had a positive, significant correlation with self-regulated learning both as a dimension and with each of its factors (planned learning, thoughtful learning, and study techniques), see **Table 3**.

**Table 3:** Correlations between different variables and self-regulated learning (*n* = 911).

Dimensions and factors	D2. Self-regulated learning	F2. Planned learning	F7. Thoughtful learning	F9. Study techniques
Deep learning approach	0.369^∗∗^	0.315^∗∗^	0.375^∗∗^	0.218^∗∗^
Surface learning approach	-0.501^∗∗^	-0.432^∗∗^	-0.416^∗∗^	-0.382^∗∗^
Emotion focused	-0.008	-0.066	-0.080	0.120
Problem-focused	0.393^∗∗^	0.291^∗∗^	0.272^∗∗^	0.419^∗∗^
Total Performance	0.187^∗^	0.254^∗∗^	0.139	0.077
D3. Satisfaction with learning	0.569^∗∗^	0.420^∗∗^	0.500^∗∗^	0.422^∗∗^

*^∗^p < 0.05, ^∗∗^p <0.01.*

*Self-regulated learning* as a dimension had a significant, positive correlation with *total performance*. However, the most notable positive relationships were between *self-regulated learning,* and its factors, and *procedural performance.* A positive association relationship was found between self-regulated learning and *satisfaction with learning* as a dimension and with its different factors (satisfaction with learning and meaningful learning).

#### Regulatory Teaching and Other Learning Process Variables

*Regulatory teaching* as a dimension had a significant, positive correlation with *goals*, as well as with *general regulatory teaching* and *total self-regulation* and two of its factors (*personal goals* and *perseverance*). It is also notable that *specific regulatory teaching* and *preparation for learning* have positive, significant relationships with *personal goals*, see **Table 4**. Regulatory teaching as a dimension had a positive, significant correlation with deep approach and deep motivation, and a negative correlation with surface approach and its factors (surface motivation and surface strategy). When considering the different factors, we find positive, significant relationships between *general regulatory teaching* and *deep approach* and its components (deep motivation and deep strategy) and a negative relationship between *general regulatory teaching* and surface approach and its components; also, *preparation for learning* and *satisfaction with the teaching* had positive, significant relationships with *deep approach* and its components (*deep motivation* and *deep strategy*).

**Table 4:** Correlations between regulatory teaching and other variables (*n* = 911).

Dimensions and factors	D1. Regulatory teaching	F1. Specific regulatory teaching	F4. Regulatory assessment	F6. Preparation for learning	F8. Satisfaction with the teaching	F12. General regulatory teaching
Total SRQ	0.133	0.096	-0.099	0.103	0.025	0.191^∗^
Personal goals	0.322^∗∗^	0.235^∗∗^	0.096	0.258^∗∗^	0.152	0.304^∗∗^
Perseverance	0.069	0.058	-0.054	0.086	0.027	0.169^∗^
Decision-making	0.000	0.040	-0.142	0.042	-0.119	0.093
Learning from mistakes	0.106	0.026	0.169^∗^	0.023	0.021	0.120
Deep learning approach	0.202^∗^	0.170	0.122	0.263^∗∗^	0.211^∗∗^	0.266^∗∗^
Surface learning approach	-0.264^∗∗^	-0.170^∗^	-0.066	-0.210^∗∗^	-0.159	-0.259^∗∗^
Emotion focused	0.066	0.090	0.142	-0.032	0.084	-0.167
Problem-focused	0.273^∗^	0.226^∗∗^	0.198^∗^	0.150	0.198	0.065
D2. Self-regulated learning	0.396^∗∗^	0.359^∗∗^	0.242^∗∗^	0.267^∗∗^	0.286^∗∗^	0.264^∗∗^
Total performance	0.118^∗^	0.093	0.164^∗^	0.177^∗^	0.176^∗^	0.206^∗∗^
D3. Satisfaction with learning	0.608^∗∗^	0.515^∗∗^	0.297^∗∗^	484^∗∗^	0.527^∗∗^	0.568^∗∗^

*^∗^p < 0.05, ^∗∗^p < 0.01.*

Pearson bivariate correlation analyses showed significant, positive correlations of regulatory teaching, specific regulatory teaching, and regulatory assessment with *problem-focused coping strategies*. *Regulatory teaching* as a dimension and its different factors had a positive, significant correlation with *self-regulated learning* (dimension) and with *study techniques*. *Regulatory teaching* was also related to *planned learning* and *thoughtful learning*. *Thoughtful learning* was also found in a positive, significant association with *specific regulatory teaching*, *regulatory assessment*, *satisfaction with the teaching,* and *general regulatory teaching*.

Pearson bivariate correlation analyses showed that *regulatory teaching* as a dimension, *regulatory assessment* and *preparation for learning* had significant, positive relationships with *total performance*, *procedural performance* and *attitudinal performance*. Only *regulatory teaching* had a positive relationship with *conceptual performance*, in addition to *procedural* and *total performance*. *Regulatory teaching* and all its factors have a positive, significant correlation with *satisfaction with learning* (dimension) and with *meaningful learning*. We also found that *regulatory teaching*, *specific regulatory teaching*, *regulatory assessment*, *preparation for learning* and *general regulatory teaching* were related significantly and positively to *satisfaction with learning*.

### Structural Models

**FIGURE 2:**
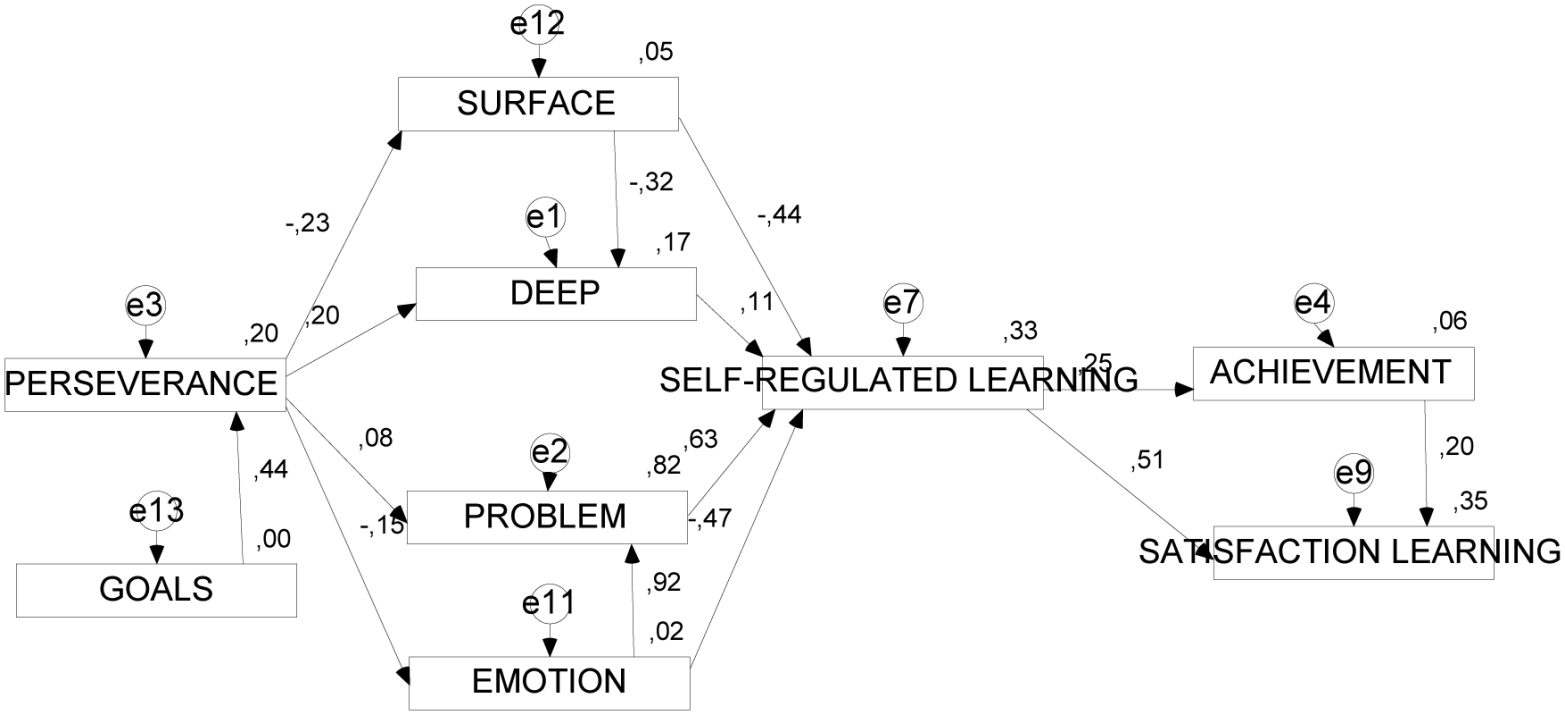
**Structural model of the effect of the presage and the process variables on performance and on satisfaction with learning (without regulatory teaching)**.

**FIGURE 3:**
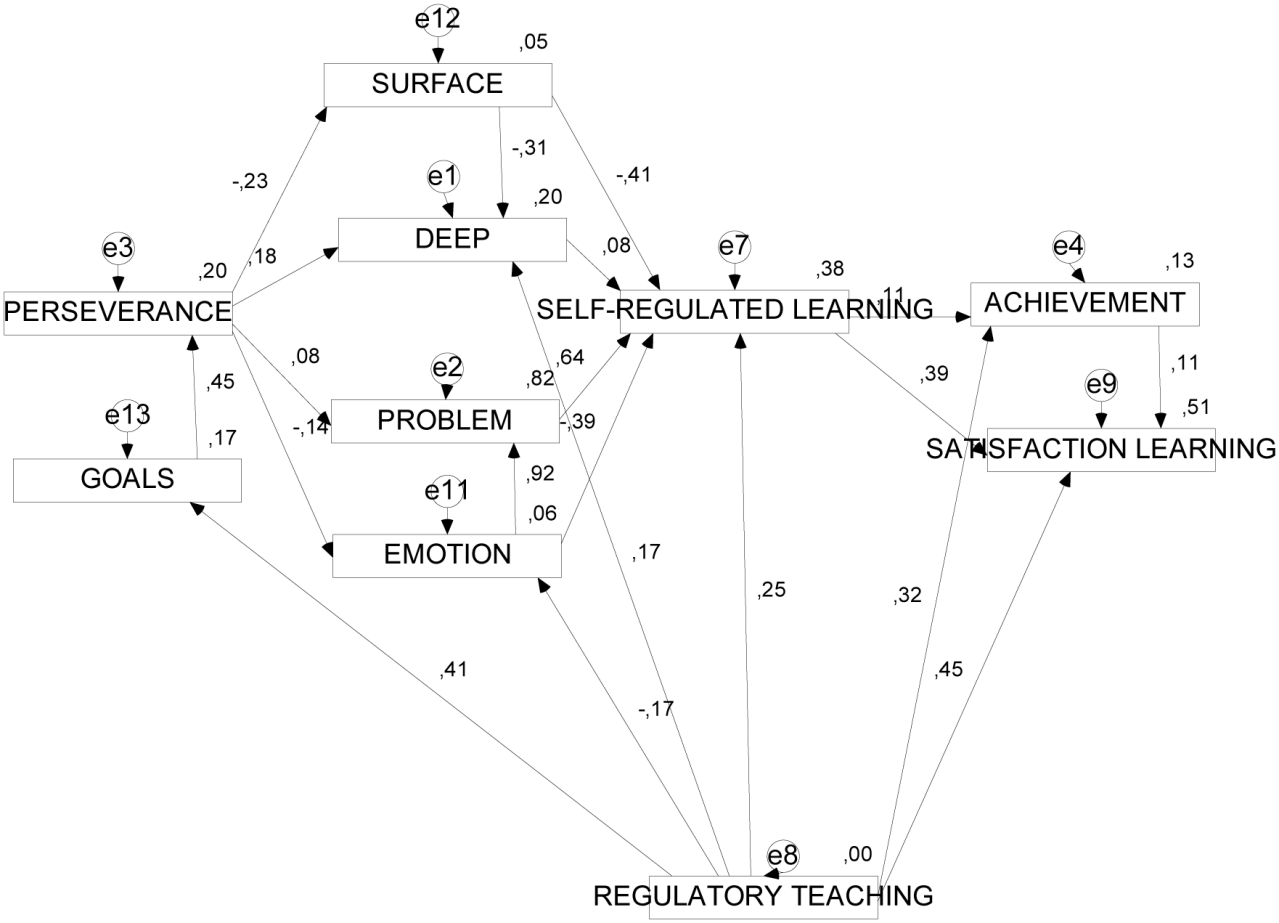
**Structural model of the effect of learning presage and process variables on performance and on satisfaction with learning (with regulatory teaching)**.

A structural path analysis showed reasonable levels of fit for the two models presented in **Table 5**, and **Figures 2** and **3**.

**Table 5:** Absolute fit statistics for the two models.

	*n*	df	χ^2^	*p*<	RMSEA	NFI	RFI	IFI	TLI	CFI
Model 1	1101	22	98,298	0.001	0.056	0.934	0.954	0.946	0.923	0.948
Model 2	1101	25	95,849	0.001	0.051	0.938	0.913	0.952	0.937	0.953


#### Model 1

Results were satisfactory in Model 1, which focuses on the learning process; indices were around 0.90 and error about 0.60. One can observe in the model how *academic performance* and *satisfaction with learning* are jointly determined by perseverance, surface approach, emotion-focused coping strategies, self-regulated learning, and regulatory teaching. Specifically, *perseverance* is the characteristic of self-regulation that, on one hand, is negatively associated with *surface approach* (SURFACE) and positively with *deep approach* (DEEP), and on the other hand, negatively associated with *emotion-focused coping strategies* (EMOTION), and positively with problem-focused coping (PROBLEM). Elsewhere, *self-regulated learning* (IATLP2) is determined negatively by *surface approach* (SURFACE) and *emotion-focused coping strategies* (EMOTION), and positively by *deep approach* (DEEP) and *problem-focused coping strategies* (PROBLEM). Finally, *self-regulated learning* (IATLP2) was a significant, positive determinant of *total performance* (GPA) and *satisfaction with learning* (SATISFACTION).

#### Model 2

Model 2 (see **Figure 3**) evaluates the same relations with the regulatory teaching variable. In this model the results were satisfactory with indices of around 0.90 and error of about 0.50. In the model one can observe how *academic performance* and *satisfaction with learning* are jointly determined by perseverance, surface approach, emotion-focused coping strategies, self-regulated learning, and regulatory teaching. Specifically, *perseverance* is the characteristic of self-regulation that, on one hand, is negatively associated with *surface approach* (SURFACE) and positively with *deep approach* (DEEP), and on the other hand, is negatively associated with *emotion-focused coping strategies* (EMOTION) and positively with problem-focused coping (PROBLEM). Elsewhere, *self-regulated learning* (SELF-REGULATED LEARNING) is determined negatively by *surface approach* (SURFACE) and *emotion-focused coping strategies* (EMOTION), and positively by *deep approach* (DEEP) and problem-focused coping strategies (PROBLEM). Finally, *self-regulated learning* (SELF-REGULATED LEARNING) was a significant, positive determinant of *total performance* (GPA) and *satisfaction with learning* (SATISFACTION). The role of *regulatory teaching* (REGULATORY TEACHING) was notable in positively determining *perseverance* (PERSEVERANCE), through *learning goals* (GOALS), *deep approach* (DEEP), *self-regulated learning* (IATLP2), *total performance* (GPA), and *satisfaction with learning* (SATISFACTION); and negatively determining the use of *emotion-focused coping strategies* (EMOTION).

## Discussion

In general, our hypotheses are confirmed, since we found the *association relations* and two *structural models* that validate the linear conceptual relationships proposed in the present study.

### Personal Self-Regulation

The association hypothesis regarding *personal self-regulation* (H1) is partly confirmed. Of the process variables analyzed (approaches to learning, coping strategies, and self-regulated learning), the *learning approaches* hypothesis has been corroborated, since the level of self-regulation (goals, perseverance, and decision-making) is positively associated with a deep approach (deep motivation and strategy), in consonance with prior evidence (Berbén, 2005). Regarding *coping strategies*, the hypothesis is partially confirmed, since level of self-regulation showed a negative association with emotion-focused strategies, but did not show the hypothesized positive association with problem-focused strategies. We would also note that, in analyzing the components of self-regulation, (1) the students with *perseverance* make less use of emotional venting and resigned acceptance; (2) those who make use of *decision-making* make less use of *preparing for the worst* and *fantasy distraction*; and (3) those who use *learning from mistakes* make less use of the *emotional venting and isolation* strategy. One noteworthy result was the positive relationship found between self-regulation (total, goals, perseverance, and learning from mistakes) and *help for taking action*. This may be due to the fact that this emotion-focused strategy has more of a cognitive nature and not as much an affective-emotional nature as other strategies of this type. De la Fuente and Cardelle-Elawar (2011) also found a negative association between personal self-regulation and emotion-focused strategies. The lack of studies on this variable within educational contexts makes it impossible for us to offer more comparison with results from previous studies.

As for *self-regulated learning*, students with self-regulation (total, goals, perseverance, and learning from mistakes) also have self-regulated learning, a result that confirms our hypothesis. If we look at the components of both variables, we see that all the components of self-regulation (total, goals, perseverance, decision-making, and learning from mistakes) increase the probability of planned learning, and also that certain components of this presage variable (total, goals, perseverance, and learning from mistakes) lead to more thoughtfulness in learning. This result, though expected, is a novel one, confirming our idea that *personal self-regulation* is a *presage* variable worth taking into account, due to its effect in the product phase of learning. Obviously, this is most strongly manifest in *self-regulated learning,* a result that is consistent with the limited prior research in this regard (Berbén, 2008).

Second, the hypothesis concerning the influence of personal self-regulation on *product* variables (satisfaction with learning and academic performance) is confirmed. With reference to *satisfaction with learning*, personal self-regulation (total, goals, perseverance, and learning from mistakes) is associated with both self-regulated learning (D3) and meaningful learning (F10). It is also important that goals and perseverance increased the likelihood of students’ satisfaction with the learning process (F3). As for performance, students with goals and perseverance obtain better total, procedural, and attitudinal performance. This result is consistent with the premise that procedural and attitudinal performance have the greatest association with self-regulation, while learning approach is more associated with conceptual performance (De la Fuente et al., 2008). Again, we stress the need for more studies in this direction, due to the association of personal self-regulation with process variables (learning approaches, coping strategies, and self-regulated learning in university students) and product variables (satisfaction with learning and performance).

In the *first structural model*, we see that goal-setting has a positive effect on perseverance, and the latter in turn influences learning approaches and coping strategies. Perseverance negatively predicts a surface approach, which in turn influences the deep approach, and it predicts less use of emotion-focused strategies, which influences the use of problem-focused strategies. The above variables have an effect on self-regulated learning. Specifically, the surface approach has a negative effect on self-regulated learning, and emotion-focused strategies also have a negative effect on this variable. This entire compendium of variables significantly affects performance and satisfaction with learning, the latter effect being more significant. Academic performance in turn affects satisfaction with learning, consistently with prior results presented above, since students with better grades may be more satisfied with their learning.

### Regulatory Teaching as Effective Teaching

The association hypothesis regarding *regulatory teaching* (H2) is partially confirmed. Regulatory teaching and some of its components (general regulatory teaching, specific regulatory teaching, and preparation for learning) predict goal-setting as a factor of *personal self-regulation* (presage variable). However, in these university students, regulatory teaching is positively associated with a *deep approach* (deep motivation) and negatively with a *surface approach* (surface motivation and strategy). If we consider the components of regulatory teaching, we find a positive association of deep approach and negative association of surface approach with general regulatory teaching, preparation for learning and specific regulatory teaching.

As for *coping strategies* (process variable), our hypothesis is partially fulfilled, since we find that regulatory teaching positively predicts problem-focused strategies. If we analyze the factors of both variables, several results can be noted: (1) specific regulatory teaching, general regulatory teaching, preparation for learning, and satisfaction with the teaching positively predict the use of *help for taking action* (emotion-focused strategy); (2) general regulatory teaching negatively predicts use of the emotion-focused strategy *resigned acceptance*; (3) the regulatory teaching dimension as a whole predicts students’ use of *positive reappraisal* and *communication of feelings and social support*.

Our hypothesis was confirmed in the case of *self-regulated learning* (process variable). *Regulatory teaching* on the part of the teacher predicts development of self-regulated learning in the students, especially in the use of study techniques and thoughtful learning. The factors of regulatory teaching that most predict development of self-regulated learning are *specific regulatory teaching* and *satisfaction with the teaching*. As for the product variables (*satisfaction with learning* and *academic performance*), our initial hypothesis has been demonstrated, since regulatory teaching predicts *satisfaction with learning* and better *procedural*, *attitudinal,* and *total performance*. Regulatory teaching and preparation for learning are the factors of regulatory teaching that best predict these types of performance.

The scarcity of research regarding the influence of regulatory teaching on these variables does not allow us to consider these results definitive, but to call for more studies to help define the influence of this variable on personal self-regulation, learning approaches, coping strategies, satisfaction with learning, and academic performance. Certain authors (De la Fuente and Justicia, 2003, 2007; De la Fuente et al., 2012) also agree on the importance of considering this variable in further studies, affirming that there is lack of regulatory teaching and self-regulated learning, perhaps due to the absence of appropriate teacher explanations at different moments of the teaching–learning process. The *second structural model* offers us a final, very important relationship, which we have defended throughout this paper. By this we refer to the effect of *regulatory teaching* on the relationships mentioned. We wish to stress its positive effect on *satisfaction with learning*, on *goal setting* and on *regulatory teaching*, and its negative effect on emotion-focused strategies (regulatory teaching predicts less use of emotion-focused strategies). All the aspects of regulatory teaching mentioned in the empirical model are characteristic of *effective teaching* (Roehrig and Christesen, 2010; Roehrig et al., 2012).

## Conclusion

The results of this study are consistent with another recent research report (De la Fuente et al., 2014). Using a linear or structural methodology, to the extent that the results permit, regulatory behavior in teaching (as a component of effective teaching) had positive effects on personal self-regulation, on approach to learning, on coping strategies, on self-regulated learning, as well as on learning satisfaction and performance. However, no positive predictive structural relationship could be established between regulatory teaching and problem-focused strategies, nor a negative relationship with a surface approach to learning. These results are consistent with prior research, which tends to more easily find the negative effects of the surface approach (Berbén, 2008) and of emotion-focused strategies (Zapata, 2013).

Despite the contribution of new results, this investigation also has its *limitations*, which should be avoided in future studies. The first limitation is due to the lack of other comparable research results that refer to our core study variables: personal self-regulation, coping strategies, and regulatory teaching. Especially in the case of personal self-regulation and of coping strategies, as we as have seen throughout this study, these variables have been studied mostly in clinical contexts. For this reason, the results obtained here are still tentative; nascent research leads us to be cautious in accepting conclusions with these variables. In any case, certain changes can be proposed for validating certain constructs, such as in the case of coping strategies: some of the factors of emotion-focused strategies may need to be redefined, particularly in the case of *help for taking action*, which has generated so much controversy in the present discussion.

Another limitation has to do with *sample attrition* in some of the analyses, since not all the students completed all of the questionnaires. With respect to *gender,* not all the students reported their sex, for this reason there was sample loss in some analyses. Future investigations should insist on the importance of completing this data point.

### Implications and Future Research

Before bringing this paper to a close, we must insist on the possible *utility of these findings for educational practice,* and stress certain general ideas that would serve for continuing this line of research. First, the personal self-regulation results raise a great many interesting questions for educational research, inasmuch as this variable has influenced and mediated other variables in the teaching–learning process. As other authors state (Karoly et al., 2005), progress in understanding the causal relationships between personal and academic self-regulation is of interest to educational practice if we wish to improve both capacities, not only at university but also in pre-university stages of education, in order to prepare students and make their transition to university easier and more satisfactory. Training in these self-regulating and coping behaviors can equip students with the needed skills that are common to both self-regulated learning and to self-regulating addictive behaviors such as the use of alcohol, tobacco, and other drugs that affect not only the student’s health but also his or her academic performance. A healthy student is more likely to obtain greater success in both academic studies and in their professional career, and less likely to fall into academic failure, with its high incidence in our day. Continuing in this line of work, students should also be equipped with the different coping strategies that can be used, particularly those that best predict success and a reduction in the academic stress that is so prevalent in our new context of Higher Education, where students must take a more active, regulating role, and where they must handle a large workload.

Second, and finally, an effort is needed to *promote and provide favorable conditions for quality teaching–learning environments* that encourage deep learning, and also to equip teachers with the necessary skills for practicing regulatory teaching in university contexts. This intervention has great importance, due to the influence of regulatory teaching, as found in our research results on presage, process and product variables. Interventions along the lines discussed here can lead to quality education, where effective teaching is fostered and where students have greater skills and competencies for facing stress in the university context. Traditionally, the student’s learning process has been analyzed exclusively from a perspective of cognitive and motivational processes. The time has come to thoroughly address emotional processes and their direct repercussions on learning, as well as the side effects of the university teaching–learning process on health processes, approaching all this predominantly from the Educational Psychology perspective.

## Conflict of Interest Statement

The authors declare that the research was conducted in the absence of any commercial or financial relationships that could be construed as a potential conflict of interest.
